# Research Progress on ^18^F-Labeled Agents for Imaging of Myocardial Perfusion with Positron Emission Tomography

**DOI:** 10.3390/molecules22040562

**Published:** 2017-03-30

**Authors:** Tiantian Mou, Xianzhong Zhang

**Affiliations:** 1 Department of Nuclear Medicine, Beijing Anzhen Hospital, Capital Medical University, Beijing 100029, China; mtt207@163.com; 2 State Key Laboratory of Molecular Vaccinology and Molecular Diagnostics & Center for Molecular Imaging and Translational Medicine, School of Public Health, Xiamen University, Xiamen 361102, China

**Keywords:** positron emission tomography, ^18^F-labeled radiotracers, myocardial perfusion imaging, mitochondrial complex-1, lipophilic cations

## Abstract

Coronary artery disease (CAD) is the leading cause of death in the world. Myocardial perfusion imaging (MPI) plays a significant role in non-invasive diagnosis and prognosis of CAD. However, neither single-photon emission computed tomography nor positron emission tomography clinical MPI agents can absolutely satisfy the demands of clinical practice. In the past decades, tremendous developments happened in the field of ^18^F-labeled MPI tracers. This review summarizes the current state of ^18^F-labeled MPI tracers, basic research data of those tracers, and the future direction of MPI tracer research.

## 1. Introduction

Though the treatments of coronary artery disease (CAD) have seen prominent improvements over the past decades, CAD is still the leading cause of death in the world. Single-photon emission computed tomography (SPECT) myocardial perfusion imaging (MPI), using radiotracers such as ^99m^Tc-sestamibi, ^99m^Tc-tetrofosmin and ^201^Tl, is the commonly used, standard, and non-invasive clinical screening tool for detecting CAD, risk stratification, and guidance of therapeutic interventions. Its sensitivity and specificity for detecting significant coronary stenosis was 87% and 73%, respectively, in a meta-analysis involving 4480 subjects [1]. However, the application of SPECT MPI is limited for the following reasons: inferior spatial and temporal resolution, incapability of absolute quantification, non-uniform attenuation correction and high uptake in the organs adjacent to the heart [2]. 

PET isotopes emit positrons. When a positron meets a nearby electron, they annihilate each other and emit two 511-keV photons in opposite direction (discharged at 180° to each other). Only the coincidental detection of two 511-keV photons can be recorded in PET scanners and reconstructed into PET images [3]. Compared with SPECT, PET technology offers a better resolution and effective correction of photo-attenuation and scatter [4], leading to absolute quantification of regional myocardial blood flow and coronary flow reserve [5]. Besides that, patients are exposed to less radiation owing to the short half-lives of positron isotopes [6,7]. Hence, the need for and use of PET applications in healthcare facilities is increasing tremendously [3,8,9,10]. In 2016, the American Society of Nuclear Cardiology and the Society of Nuclear Medicine and Molecular Imaging published a joint position statement on the clinical indications for the significant underutilization of myocardial perfusion PET in America [11,12]. ^13^N-NH_3_, ^82^Rb, and ^15^O-H_2_O are representative clinical PET MPI tracers [13]. As the half-lives of ^15^O-H_2_O and ^13^N-NH_3_ are very short (2 and 10 min, respectively) [14], on-site cyclotrons are required. ^82^Rb has a very short half-life (75 s) but can be conveniently supplied from a bedside generator. However, it has a low myocardial ejection fraction at high flow rates. Hence, it is imperative to develop novel and preferable PET MPI agents.

Compared with the aforementioned isotopes, ^18^F has much shorter average positron range (1.03 nm), which results in better spatial resolution and contrast. It also has a longer half-life (110 min), so ^18^F-labeled radiotracers can be supplied at regional cyclotrons and allow treadmill exercises [15]. Thus, the development of ^18^F-labeled MPI agents becomes a hot topic of interest for many researchers. Numerous novel agents have been synthesized and studied in the past decade. Especially a series of reports about ^18^F-flurpiridaz evidently enhanced researchers’ confidence on the future of PET MPI agents. Several published reviews have summarized the characteristics of PET MPI agents and compared them with ^13^N-NH_3_, ^82^Rb, and ^15^O-H_2_O [14,16,17,18,19]. However, most of them focused on the MPI agents in clinic status. In this study, we summarized ^18^F-labeled radiotracers in both clinical and preclinical status.

According to the chemical structures and mechanism, ^18^F-labeled MPI agents under investigation can be divided into two types: lipophilic cations and analogues of mitochondrial complex-1 (MC-1) inhibitors. Herein, we compared the characteristics, mechanism, and research status of those different tracers.

## 2. Lipophilic Cations

**Figure 1:**
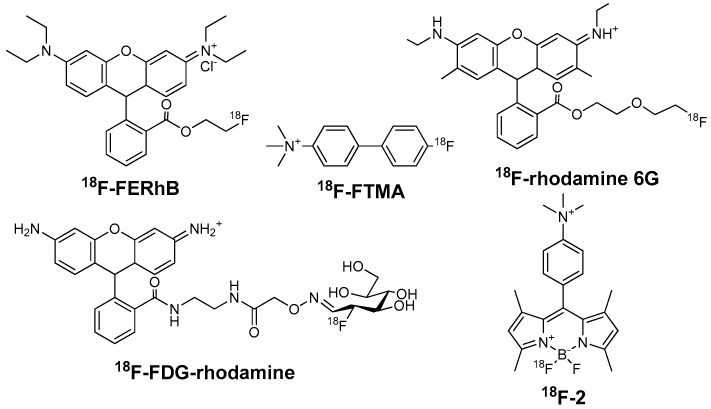
The structures of several ^18^F-labeled ammonium lipophilic cations.

**Figure 2:**
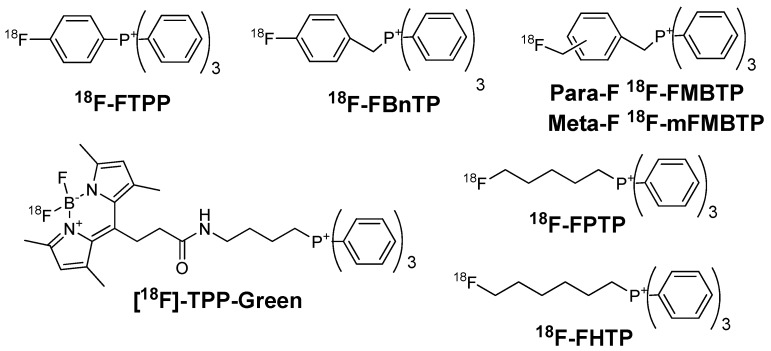
The structures of several ^18^F-labeled phosphonium lipophilic cations.

Mitochondria take up 20–30% of the myocardial intracellular volume, making it an ideal target for MPI. The activation energy of lipophilic cations for moving through hydrophobic barrier of a biological membrane is far lower than that of other cations. Hence, lipophilic cations can pass through phospholipid bilayers of mitochondria without requiring a specific uptake mechanism [20]. Because of delocalized positive charge of lipophilic cations, they accumulated substantially in the mitochondria in a membrane potential-dependent manner [21]. Myocardial ischemia can cause cell death. Loss of mitochondrial membrane potential is an early event in cell death [22]. Lipophilic cations can be used to detect myocardial abnormalities because the uptake of which is sensitive with mitochondrial voltage. Lipophilic cations for MPI include two types such as ammonium cations (Figure 1) and phosphonium cations (Figure 2).

### 2.1. Ammonium Cations

#### 2.1.1. 4-^18^F-Fluorotri-*N*-Methylanilinium Iodide (^18^F-FTMA)

Studenov et al. synthesized four ^18^F-labeled ammonium salts, represented by ^18^F-FTMA [23]. The studies of acetylcholinesterase (AcChE) inhibition suggested that the myocardial accumulation of ^18^F-FTMA was probably due to the binding with myocardial AcChE (K_i_: 46–49 μM). The biodistribution study in mice revealed that ^18^F-FTMA had low myocardial uptake and heart/liver ratio (<0.5 during 5–60 min post injection (p.i.)). Hence, it had a limited potential for MPI.

#### 2.1.2. ^18^F-Labeled Rhodamines

Rhodamine can accumulate in the mitochondria in proportion to mitochondrial membrane potential. ^18^F-FERhB was developed for MPI because the lead unlabeled compound rhodamine-123 could accumulate well in the heart of mouse [24]. The imaging capability of ^18^F-FERhB was related to its stability. For instance, 71% of ^18^F-FERhB got hydrolyzed in mouse serum at 2 h p.i., leading to poor myocardial uptake in the microPET image of a mouse. Whereas 86% of ^18^F-FERhB was still intact in rat serum at the same condition, resulting in considerable increase of myocardial uptake in rats [25]. In the biodistribution study of rats, the myocardial uptake (2.06 ± 0.61% ID/g at 60 min p.i.) was over twice the liver uptake and over 25 times the blood uptake. However, the myocardial image of the rat indicated that the uptake in heart and liver was approximately equal so it might not be a competitive MPI agent. Researchers supposed that ^18^F-FERhB might have better performance in human, since it had a better stability in human serum.

Maddahi et al. used ^18^F-FDG as the radiointermediate, and reported the preclinical evaluation of ^18^F-FDG-rhodamine [26]. ^18^F-FDG-rhodamine had a good stability in human plasma in vitro. The heart uptake of ^18^F-FDG-rhodamine was 11.24 ± 1.97% ID/g in rats, which was nearly 4 times higher than other radiofluorinated rhodamine analogues. The low lipophilic characteristic (log P = −1.64 ± 0.03) leads to the low liver uptake. The heart/liver ratio was 21.20 at 60 min p.i. The myocardial extraction of ^18^F-FDG-rhodamine was 27.63 ± 5.12% during the first 15 min of perfusion period, which was higher than ^99m^Tc-MIBI (15 ± 1%) and lower than ^201^Tl (30 ± 5%). Besides that, Maddahi et al. mentioned that ^18^F-FDG-rhodamine hydrolysed in vivo in mice as ^18^F-FERhB. They suggested that mice might not be the suitable animal models for the tests of rhodamine-related compounds [26].

On the other hand, Bartholomä et al. developed a range of different rhodamine cores (rhodamine 6G, rhodamine 101, and tetramethylrhodamine) labeled with ^18^F. They used various rhodamine lactones as the precursors and used ^18^F-fluorodiethylene glycol ester as the prosthetic group [27]. Rhodamine 6G could locate in the mitochondria of isolated rat cardiomyocytes and had superior pharmacologic properties than others. Further first-in-human clinical studies with ^18^F-rhodamine 6G are on the way. So far, there is no follow-up report published in literature concerning its stability and clinical application in human.

#### 2.1.3. ^18^F-Labeled BODIPY Derivatives

Boron-dipyrromethene (BODIPY) is a class of fluorescent dyes. It contains dipyrromethene and a disubstituted boron atom. BODIPY derivatives accumulate in mitochondria in a mitochondrial membrane potential-dependent manner [28]. They can be labeled with ^18^F by SnCl_4_-promoted ^18^F-^19^F isotopic exchange in aqueous solutions. Li et al. reported a series of ^18^F-labeled BODIPY derivatives [29,30,31]. Most of them had low heart/liver ratio, which limited their potential as MPI agents. 10-(4-(trimethylammonio)phenyl)-5-fluoro-5-^18^F-fluoro-1,3,7,9-tetramethyl-5*H*-dipyrrolo[1,2-c:2′,1′f][1,3,2] diazaborinin-4-ium-5-uide (^18^F-2) was the representative compound [31]. The heart uptake and heart/liver ratio of ^18^F-2 was 8.75% ± 1.04% ID/g and 2.19 ± 0.42 at 60 min p.i. in mice. In addition, all seven reported agents showed certain bone uptake (from 1.03 ± 0.21 to 2.74 ± 0.15% ID/g at 60 min p.i. in mice), indicating the decomposition of agents in vivo. So far, ^18^F-labeled BODIPY derivatives haven’t showed remarkable biological properties. However, since BODIPY can be developed as PET/optical dual-modality agents, and the labeling method is mild and simple, novel BODIPY derivatives may be competitive in the future.

### 2.2. Phosphonium Cations

Phosphonium cations were studied much widely than ammonium cations. Most of them showed superior properties in both MPI and detection of apoptosis. The modification of phosphonium cations was focused on the labeling methods and biological properties. The representative agents were ^18^F-FBnTP, ^18^FTPP, ^18^F-FPTP, ^18^F-FHTP, and ^18^F-mFMBTP.

#### 2.2.1. ^18^F-Fluorobenzyl Triphenyl Phosphonium (^18^F-FBnTP) Cation

^18^F-FBnTP is the incipient ^18^F-labeled phosphonium cation [32]. In the imaging study of dogs, it showed notable initial uptake and prolonged retention in the myocardium [21]. The clearance from blood pool was rapid (half-life: 19.5 ± 4.4 s), reaching 26.2 ± 7.8% and 13.4 ± 6.3% of activity in the left ventricular wall at 5 and 10 min, respectively. At 60 min p.i., the heart/blood, heart/lung, and heart/liver ratios were 16.6:1, 12.2:1, and 1.2:1, respectively. The detailed anatomy of the heart including the papillary muscle and the left and right atria could be easily recognized because of low background activity in combination with extensive uptake and prolonged retention in the myocardium. ^18^F-FBnTP was eliminated mainly via kidneys than hepatobiliary tract. It is sensitive in detecting small flow defects with similar accuracy all over the myocardium, including the inferior aspect adjacent to the liver.

**Figure 3:**
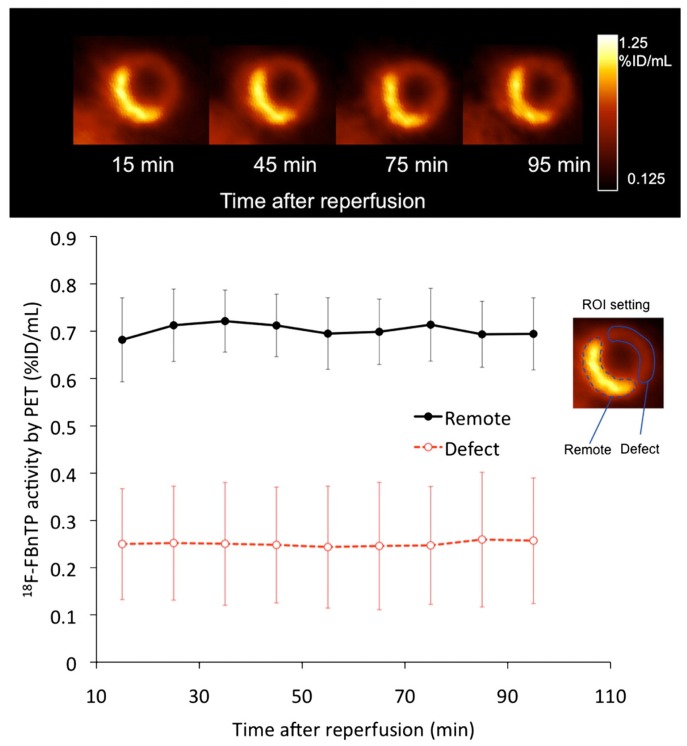
Representative short-axis small-animal PET images of ^18^F-FBnTP at different time points after tracer administration during short-term occlusion, followed by reperfusion. Graph shows mean uptake in defect and remote area. %ID = percentage injected dose; RO = region of interest [34].

Compared with ex vivo tissue staining, the ischemic area after coronary occlusion assessed by PET was 16% smaller [33]. Compared with ^99m^Tc-tetrofosmin, the accuracy of ^18^F-FBnTP was far better in the determination of mild and severe stenosis. In addition, ^18^F-FBnTP showed stable delineation of the ischemic area with no appreciable washout or redistribution (Figure 3) compared with ^201^Tl [34]. At the beginning, the main limitation of ^18^F-FBnTP was its radiosynthesis. The first report of radiosynthesis took four steps (82 min) with poor radiochemical yield (6%), making it inconvenient for clinical application [21]. Since then, researchers have devoted their efforts to simplifying the synthesis [35,36]. In 2016, Zhang et al. reported a one-step synthesis of ^18^F-FBnTP by a copper-mediated ^18^F-fluorination reaction with a pinacolyl arylboronate precursor [37]. The total radiochemical yield was 60 ± 18% without correction. This was a huge step for the promotion of ^18^F-FBnTP.

#### 2.2.2. (4-^18^F-Fluorophenyl)triphenylphosphonium (^18^F-FTPP) Cation

Since ^18^F-FBnTP exhibited remarkable biological properties for MPI, researchers tried to develop further novel phosphonium cations with higher radiochemical yield and better biological properties. Zhen et al. reported ^18^F-FTPP (also named ^18^F-TPP) as a potential MPI agent [38]. ^18^F-FTPP was originally developed for tumor imaging. However, besides tumor accumulation, it also showed significant myocardial uptake.

The radiochemical yield of ^18^F-FTPP was 10–15% at end of synthesis (EOS). The biodistribution and imaging studies in rats indicated a rapid accumulation of ^18^F-FTPP in the heart (1–2 min) with stable retention for at least 1 h [39]. The heart uptake of ^18^F-FTPP (1.51 ± 0.04% ID/g in rats at 30 min p.i.) was similar with ^99m^Tc-MIBI. The clearance of ^18^F-FTPP from non-target tissues was fast, resulting in high heart/blood ratios (75:1) and favorable heart/lung (4:1) and heart/liver ratios (8:1). In the coronary occlusion model of rabbits, ^18^F-FTPP showed diminished activity in the area of left anterior descending occlusion. The heart uptake of ^18^F-FTPP in the occluded myocardial regions of interest was comparable to that of ^13^N-NH_3_. Compared with ^18^F-FBnTP, ^18^F-FTPP distributes its positive charge over all four aryl groups attached to the phosphorus atom and generates a more uniform lipophilic cationic sphere. However, its potential still needs to be extensively evaluated in further animal studies.

#### 2.2.3. ^18^F-Labeled Fluoroalkylphosphonium Derivatives

Kim et al. prepared a series of tracers, such as (5-^18^F-fluoropentyl)triphenylphosphonium cation (^18^F-FPTP), (6-^18^F-fluorohexyl)triphenylphosphonium cation (^18^F-FHTP), (2-(2-^18^F-fluoro-ethoxy)ethyl)triphenylphosphonium cation (^18^F-FETP), and (2-(2-^18^F-fluoroethoxy)ethyl) tris(4-methoxyphenyl)phosphonium cation (^18^F-FETMP). ^18^F-FPTP, ^18^F-FHTP, and ^18^F-FETMP used ^18^F-fluoroalkyl-4-methylbenzenesulfonate as radiointermediates [32,40,41]. The radiochemical yields of those tracers were 10–20%, which was similar with that of ^18^F-FTPP.

**Figure 4:**
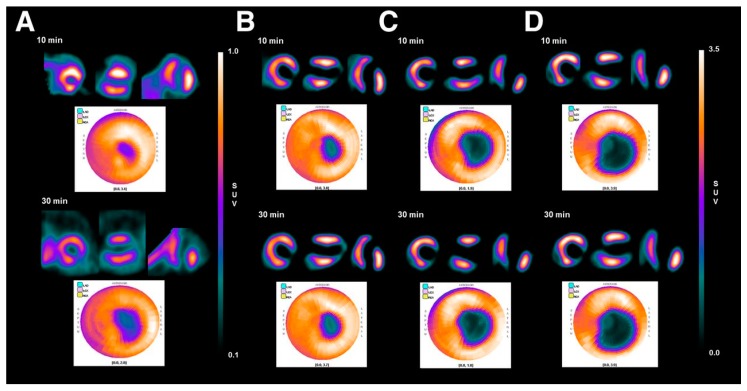
Short-, vertical long-, and horizontal long-axis and polar map images of ^13^N-NH_3_ (**A**); ^18^F-FPTP (**B**); ^18^F-FHTP (**C**); or ^18^F-FETP (**D**) in each representative animal. Data were collected between 0–10 and 20–30 min after radiotracer injection (37 MBq). SUV = standardized uptake value [42].

^18^F-FPTP and ^18^F-FHTP have similar structures and physicochemical properties. The biodistribution of these two tracers are similar in most of the organs except liver. The liver clearance rate of ^18^F-FHTP was much faster than that of ^18^F-FPTP in mice, resulting in over twice heart/liver ratios of ^18^F-FHTP (25.53 ± 5.88 at 2 h p.i.) than that of ^18^F-FPTP (10.72 ± 2.17 at 2 h p.i.). The heart/blood ratios of ^18^F-FHTP were also admirable (138.61 ± 8.10 at 2 h p.i.). In the imaging studies of rats, the myocardial uptakes of both ^18^F-FHTP and ^18^F-FPTP were stable at a constant level for up to 1 h p.i. [40]. Kim et al. compared ^18^F-FPTP, ^18^F-FHTP, and ^18^F-FETP with ^13^N-NH_3_ in rat models (Figure 4) [42]. They found that the first-pass extraction fraction values of these four radio-agents are comparable at low flow velocity (0.5 mL/min), but ^18^F-FPTP, ^18^F-FHTP, and ^18^F-FETP had significantly higher extraction fractions than ^13^N-NH_3_ at higher flow velocity (4.0, 8.0, and 16.0 mL/min, *p* < 0.05). Small animal PET images with ^18^F-FPTP demonstrated an excellent image quality with a clear delineation of the borders of defects, which was consistent with the size validated by 2,3,5-triphenyltetrazolium chloride (TTC) staining (*r*^2^ = 0.92, *p* < 0.001) [40].

The structure of ^18^F-FETMP contains methoxy and ethoxy groups to attenuate the lipophilicity. However, the liver uptake of ^18^F-FETMP was much higher than that of ^18^F-FPTP and ^18^F-FHTP, leading to much slower liver clearance [41]. Researchers supposed that the clearance of these radiotracers from liver was dependent on both lipophilicity and the functional groups of the compounds [43]. It is worth mentioning that the studies of these radiotracers in larger animals haven’t been reported yet. Hence, their perspectives for MPI need further investigations with big animal models.

#### 2.2.4. 4-(^18^F-Fluoromethyl)benzyltriphenyl Phosphonium (^18^F-FMBTP) Cation and (3-(^18^F-Fluoro-methyl)benzyl)trisphenylphosphonium (^18^F-mFMBTP) Cation

**Figure 5:**
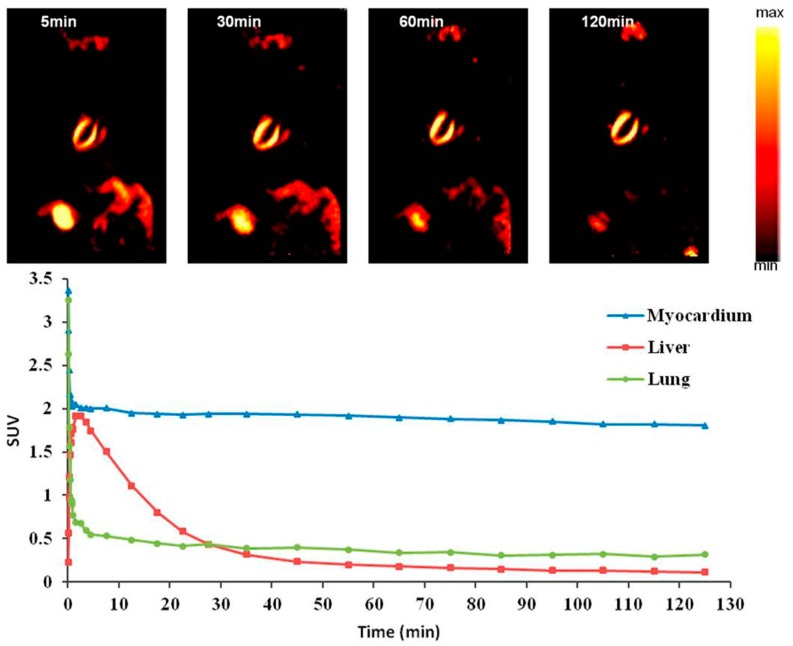
Coronal microPET images in a normal rat. The heart was visible with excellent ratios of heart/liver and heart/lung, and fast clearance from small intestine at 5, 30, 60, and 120 min after iv injection of [^18^F]mFMBTP, respectively. Time–activity curves generated from dynamic PET images. [^18^F]mFMBTP accumulated specifically in the heart. The [^18^F]mFMBTP had excellent heart/liver and heart/lung ratios and in liver and lung was washed out rapidly but was retained in the myocardium for the whole time [44].

Zhao et al. reported the preparation of ^18^F-FMBTP and ^18^F-mFMBTP using another kind of radiointermediates, *p*- or *m*-substituted 1-halomethyl-^18^F-fluoromethylbenzenes [44]. They improved the reaction condition and increased the radiochemical yields of *p*- or *m*-radio-intermediates from 12% and 26% to 85% and 92%, respectively. The radiochemical yields of final radiotracers ^18^F-FMBTP and ^18^F-mFMBTP were ~50%, which were far higher than ^18^F-FHTP (10%–20%). ^18^F-mFMBTP had good retention in the myocardium (26.82 ± 3.46% ID/g at 120 min p.i.) and faster liver clearance (1.02 ± 0.2% ID/g at 120 min p.i.). The heart/liver, heart/lung, and heart/blood ratios of ^18^F-mFMBTP were 26.25, 9.97, and 83.98 at 120 min p.i., respectively, which was comparable with ^18^F-FPTP and ^18^F-FHTP. There was certain uptake in the bone of mice. However, fortunately no obvious bone uptake was observed in the PET images of rats (Figure 5) and dogs (the heart/bone ratios of ^18^F-mFMBTP in dogs were >10 in 120 min p.i.). MicroPET studies of ^18^F-mFMBTP resulted in high contrast images with sustained prominent myocardium uptake and markedly low liver and lung uptake up to 120 min p.i. Furthermore, the heart/liver and heart/lung standardized uptake value (SUV) ratios of ^18^F-mFMBTP in dogs were calculated as 2.83 and 15.19 at 30 min p.i., and 7.76 and 35.28 at 120 min p.i., respectively. On the other hand, other organs and tissues had low background uptake because of excellent metabolic properties of the compound.

In brief, *p*- or *m*-substituted 1-halomethyl-^18^F-fluoromethylbenzenes can evidently raise the radiochemical yields of phosphonium cations [45]. Since there was certain uptake of ^18^F-FMBTP and ^18^F-mFMBTP in the bone of mice, the stability and uptake of these radiotracers in bones and other organs of big animals still need to be studied carefully.

Besides the agents above, Yuan et al. used the triphenylphosphonium group as a mitochondrial delivery vehicle. They connected the triphenylphosphonium group with BODIPY Green, and developed ^18^F-TPP-Green [46]. In summary, there is an enormous progress in the development of ^18^F-labeled phosphonium cations, especially in the radiosynthesis. Varieties of labeling methods have been used for the preparation of ^18^F-labeled phosphonium cations. The radiochemical yield has been increased from 6% to 60%. In the meantime, some novel ^18^F-labeled phosphonium cations exhibit favorable metabolic properties in the preliminary research. However, most of them have not been studied thoroughly. Additional researches are required to understand the implications of ^18^F-labeled phosphonium cations in MPI of humans.

## 3. Analogues of MC-1 Inhibitors

**Figure 6:**
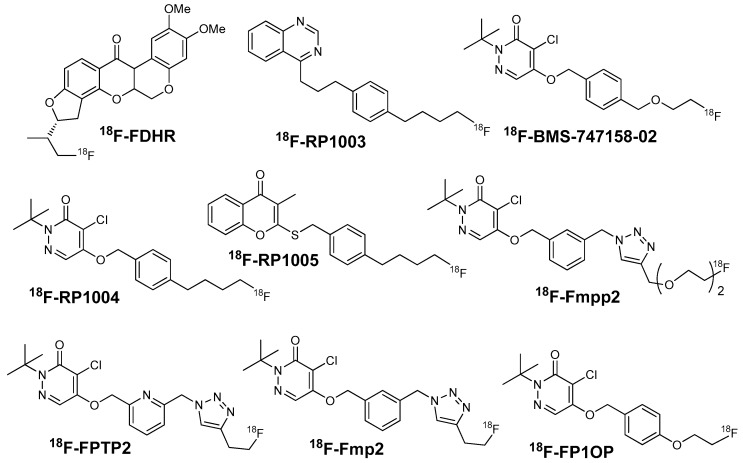
The structures of several ^18^F-labeled mitochondrial complex-1 inhibitors.

MC-1 is the first enzyme of the electron transport complexes. It locates in the inner mitochondrial membrane [47]. MC-1 has an extremely complex structure with over 40 subunits and a molecular mass of approximately 1000 kD [45]. The inhibitors of MC-1 such as rotenone, quinazoline, and pyridazinone can specifically bind to MC-1 and accumulate in the mitochondria. The heart uptake of these compounds is correlated with the myocardial blood flow. Hence, MC-1 inhibitor analogues are developed for MPI (Figure 6).

### 3.1. ^18^F-Fluorodihydrorotenone (^18^F-FDHR)

Rotenone is a neutral lipophilic compound that can inhibit the activity of MC-1. It is widely used as an insecticide. Marshall et al. found that ^125^I-iodorotenone was superior to ^99m^Tc-sestamibi as a blood flow tracer in the isolated rabbit heart [48]. Later, they prepared ^18^F-fluorodihydrorotenone (^18^F-FDHR) [49]. In the study of an isolated rabbit heart, ^18^F-FDHR was more closely related to coronary flow than ^201^Tl. Researchers considered that ^18^F-FDHR was a better blood flow tracer than ^201^Tl. Unfortunately, there is no follow-up study yet to confirm the tracer properties of ^18^F-FDHR in animal models.

### 3.2. (2-Tert-butyl-4-chloro-5-[4-(2-^18^F-fluoroethoxymethyl)-benzyloxy]-2H-pyridazin-3-one (^18^F-Flurpiridaz)

Yu et al. developed a series of ^18^F-RP1003, ^18^F-RP1004, ^18^F-RP1005, and ^18^F-flurpiridaz (previously named as BMS-747158-02) radiotracers based on different kinds of MC-1 inhibitors [50]. Among them, pyridaben is considered as the best lead compound. ^18^F-flurpiridaz, a pyridaben analogue specific binding with the PSST subunit of MC-1, is the most promising MPI agent for clinical implementation [51].

^18^F-flurpiridaz has now been in Phase III clinical trials [52]. The recent reviews considered it as the most promising tracer for MPI [16,17,53]. The biodistribution studies of ^18^F-flurpiridaz in mice showed a significant myocardial uptake and good retention properties (9.5 ± 0.5% ID/g at 60 min p.i.). The heart/liver and heart/lung ratios were 8.3 and 14.1, respectively [54]. In the imaging studies of mouse, rat, rabbit, pig, and non-human primate models, ^18^F-flurpiridaz demonstrated excellent properties with distinct visualization of the right and left ventricular myocardium and contrast between the heart and surrounding organs [51,54,55,56,57]. In coronary occlusion and ischemia/reperfusion models of rats, the images of ^18^F-flurpiridaz displayed clear and stable delineation in the non-perfused segments of myocardium. Sherif et al. demonstrated that the uptake of ^18^F-flurpiridaz in the defect area of myocardium determined by PET was closely correlated with TTC staining (*r* = 0.89, *p <* 0.01) [57]. Furthermore, the uptake of ^18^F-flurpiridaz did not change at different time points of acquisition in the infarct area of rats produced by ligating the left anterior descending artery.

**Figure 7:**
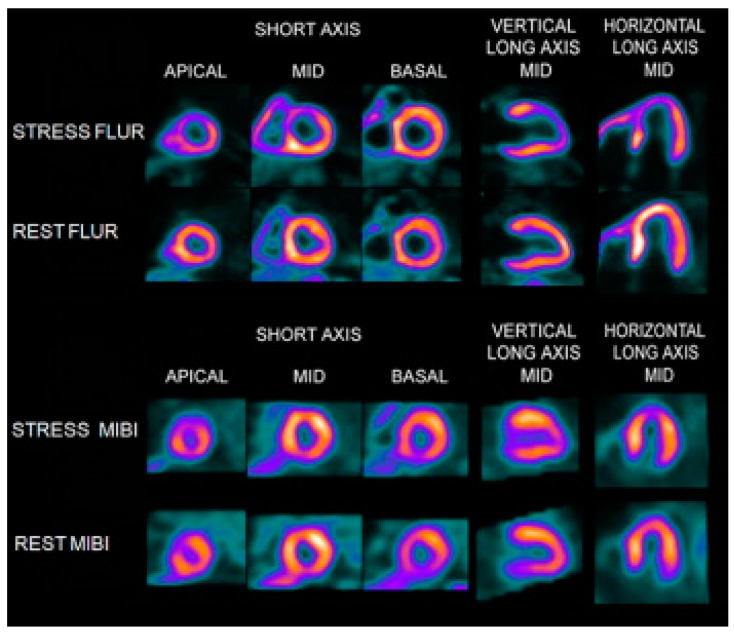
The ^18^F-flurpiridaz PET (**top**) and MIBI SPECT (**bottom**) images from an 82-year-old man with shortness of breath and an occluded native proximal left anterior descending (LAD) coronary artery and an occluded left internal mammary graft to the LAD and no other significant native CAD. The ^18^F-flurpiridaz images show a severe reversible perfusion defect throughout the territory of the occluded proximal LAD, whereas the MIBI images show only a moderate perfusion defect in the distal LAD territory (apical slices) [58].

In Phase I clinical trial in human subjects (*n* = 13), no significant adverse events related with ^18^F-flurpiridaz administration were reported [6]. The largest mean dose was absorbed by the kidneys (0.066 mSv/MBq), followed by the heart wall (0.048 mSv/MBq). The radiation dose of ^18^F-flurpiridaz is comparable to or less than that of ^18^F-FDG [6]. In a Phase II, multicenter clinical trial comprising 143 patients, ^18^F-flurpiridaz had more favorable diagnostic accuracy for evaluating multi-coronary artery stenosis, compared with SPECT MPI agents ^99m^Tc-sestamibi, ^99m^Tc-tetrofosmin, and ^201^Tl (Figure 7) [58]. In the Phase III clinical trial comprising 72 sites and 795 subjects [52], ^18^F-flurpiridaz showed a significant reduction in radiation exposure (6.1 ± 0.4 mSv) compared with SPECT (13.2 ± 3.3 mSv). In obese subjects, ^18^F-flurpiridaz showed statistically superior sensitivity, specificity, accuracy, diagnostic confidence, and image quality.

### 3.3. 2-Tert-butyl-4-chloro-5-(4-(2-^18^F-fluoroethoxy))benzyloxy-2H-pyridazin-3-one (^18^F-FP1OP) and 4-Chloro-2-tert-butyl-5-[2-[[1-[2-[2-[^18^F]fluroethoxy]ethoxymethyl]-1H-1,2,3-triazol-4-yl]methyl]phenyl-methoxy]-3(2H)-pyridazinone ([^18^F]Fmpp2)

To improve the liver clearance of pyridaben analogues such as ^18^F-flurpiridaz, Mou et al. introduced the polyethylene glycol (PEG) group into the structure and prepared ^18^F-FP1OP, ^18^F-FP2OP, and ^18^F-FP3OP [59,60]. All three tracers had low initial liver uptake (2.72 ± 0.33% ID/g, 6.14 ± 0.48% ID/g, and 2.71 ± 0.93% ID/g, respectively, at 2 min p.i.), indicating that PEG group could be an available functional group to decrease the liver uptake of those tracers. Follow-up study findings also confirmed this point of view [61,62]. 

**Figure 8:**
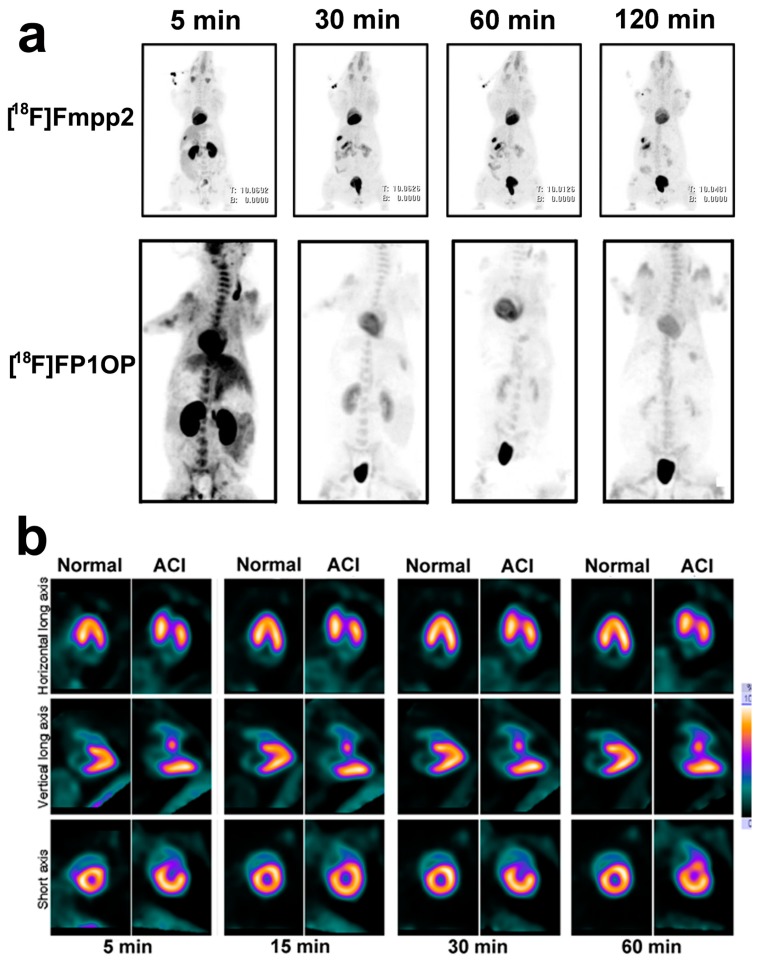
(**a**) Whole-body planar images of healthy Chinese mini swine. Images were obtained with 55 MBq of [^18^F]FP1OP [60] or 37 MBq of [^18^F]Fmpp2 [62] in 5% ethanol solution at 2, 30, 60, and 120 min after injection. B = urinary bladder; H = heart; K = kidney; (**b**) Cardiac PET images of healthy Chinese mini swine (normal) and Chinese mini swine with acute myocardial infarction (AMI). Images were obtained with 55 MBq of [^18^F]FP1OP in 5% ethanol solution at 2, 15, 30, and 60 min after injection. Arrows indicate perfusion defect sites of infarction areas (apical and anterior walls) [60].

^18^F-FP1OP had shown the prominent potential properties among those three tracers. In the imaging study in pigs, the heart/liver and heart/lung SUV ratios were 1.83 and 4.53 at 2 min p.i., 2.73 and 7.39 at 30 min p.i., and 3.03 and 8.77 at 60 min p.i., respectively. ^18^F-FP1OP can distinguish the normal myocardium, ischemic myocardium, and infarct myocardium after acute infarction (Figure 8). However, the stability of ^18^F-FP1OP in water solution is not good, which limits its further application.

Mou et al. hypothesized that the instability of ^18^F-FP1OP might due to its phenolic group [61]. Subsequently, they replaced p-substituted phenolic group with 6-methylene-2-pyridyl and 6-methylene-2-phenyl, prepared ^18^F-FPTP2 [61] and ^18^F-Fmp2 [61], respectively. Both ^18^F-FPTP2 and ^18^F-Fmp2 exhibited excellent stability in water and murine plasma, indicating the replacement of phenolic group was an effective strategy. ^18^F-FPTP2 showed a significant initial heart uptake (39.70 ± 2.81% ID/g at 2 min p.i.) and moderate retention (20.09 ± 1.93% ID/g at 60 min p.i.), indicating that a variety of aromatic rings could be used to form pyridaben analogues. This result may expand the design of pyridaben analogues for MPI.

However, the biological properties of ^18^F-FPTP2 and ^18^F-Fmp2 are not as good as ^18^F-FP1OP. Recently, they developed [^18^F]Fmpp1, [^18^F]Fmpp2 and [^18^F]Fmpp3 [62]. Among these three tracers, [^18^F]Fmpp2 exhibited the best properties. It was stable in water for at least 3 h. In the whole-body PET/CT images of mini-swine (Figure 8a), it showed excellent initial heart SUV (7.12 at 5 min p.i.) and good retention (5.75 at 120 min p.i.). The heart/liver SUV ratios were 4.12, 5.42 and 5.99 at 30, 60 and 120 min after injection, respectively. Compared with ^18^F-flurpiridaz, [^18^F]Fmpp2 has much faster liver clearance, so it may provide better quality images earlier (15–30 min p.i.). Moreover, unlike other MPI agents, the kidney uptake of [^18^F]Fmpp2 was low from 30 to 120 min p.i., which might decrease the radiation dose. The metabolic stability of [^18^F]Fmpp2 in mice, rats and Chinese mini-swine was different. In the heart of mice, 45% activities of [^18^F]Fmpp2 were metabolized at 60 min p.i. while only 13% activities of [^18^F]Fmpp2 were metabolized in both the hearts of rat and Chinese mini-swine at 60 min p.i.. Nevertheless, more studies need to be done to evaluate the potential of [^18^F]Fmpp2 in ischemic or acute myocardial infarction animal models.

Unlike ^18^F-labeled phosphonium cations, the labeling methods of ^18^F-labeled MC-1 inhibitors are similar, by substituting tosyl group with ^18^F. Radiochemical yields of ^18^F-labeled MC-1 inhibitors are, on the whole, favorable. The performance evaluation of ^18^F-labeled analogues of MC-1 inhibitors, especially ^18^F-flurpiridaz, is studied much extensively than ^18^F-labeled phosphonium cations, and thus they might be the target tracers for MPI in the coming future.

## 4. Conclusions and Perspectives

SPECT is still the first choice of MPI, especially in the developing countries. In 2012, the Chinese Society of Nuclear Medicine performed a general survey of 30 provinces regarding the status of nuclear medicine in China [63]. According to the survey data, the total SPECT examinations were more than 1.44 million cases per year. MPI studies constituted 7% of them. The total PET examinations were 0.31 million cases per year. The cardiac PET examinations constituted only 0.62% of them. In 2016, the survey data indicated that the SPECT and PET examinations in China increase to 2.1 million and 0.47 million cases per year [64]. The cardiac PET examinations constituted only 0.8% of PET examinations. We believe that the almost no PET MPI examinations in China must be due to the lack of commercialized PET agents for MPI.

However, the disadvantages of SPECT mentioned above make it difficult to satisfy the clinical application in the coming future. An ideal myocardial perfusion tracer should include the following characteristics: high myocardial extraction fraction, excellent image quality, absolute quantification of myocardial blood flow, one-day protocol for rest and stress MPI, and possibility for long-distance transportation. Due to the excellent features, ^18^F radiotracers become the most prominent isotopes for MPI. 

^18^F-flurpiridaz is the most promising MPI agent. The clinical trials with ^18^F-flurpiridaz have shown exciting results. Meanwhile, several ^18^F-labeled radiotracers such as ^18^F-FBnTP, ^18^F-FTTP, ^18^F-FHTP, and ^18^F-Fmpp2 have shown remarkable properties in preclinical studies as well. The characters of those MPI agents were brief summarized in Table 1. Many of them exhibited high heart uptake and heart/liver ratios. But most of them still need further studies to meet the criteria of clinic, such as defect delineation, polar maps reversibility, etc.

In general, lipophilic cations exhibit superior heart/liver and heart/blood ratios at early time points in small animals. For example, the heart/liver and heart/blood ratios of ^18^F-FHTP are 11.90 ± 3.37 and 71.87 ± 21.63 at 30 min p.i., respectively, in mice. Hence, clear images can be obtained early after injection of ^18^F-FHTP. However, the performances of lipophilic cations in larger animals have not yet been studied extensively. Further great efforts are needed to prove the properties of lipophilic cations in preclinical studies with larger animals and in human clinical trials. On the other hand, studies on ^18^F-labeled MC-1 inhibitors, especially pyridaben analogues, have been extended from preclinical studies involving mice to human clinical trials. Although their target/non-target ratios are not as high as lipophilic cations in mice, they are still more likely to be used in clinical practice because of prolonged retention in myocardium and low background uptake. There was certain concern about the safety of MC-1 inhibitors as the inhibition of MC-1 activity might lead to the death of animals. Fortunately, numerous studies have demonstrated that the use of MC-1 inhibitors is safe for MPI due to their extremely low chemical dose.

In addition, the studies of structure-activity relationship based on lipophilic cations or analogues of MC-1 inhibitors reveal that there is extensive scope for the modification of structures. For instance, the benzene ring of phosphonium cations can be connected with various groups and different kinds of aromatic rings can be used as the “side chain” of pyridaben. Thus, more novel ^18^F-labeled MPI tracers may be developed. The superior properties of ^18^F-labeled MPI tracers may likely increase the acceptance of cardiac PET as a routine diagnostic tool in future.

## Figures and Tables

**Table 1:** Comparison of representative ^18^F-labeled compounds as potential MPI agents.

Probes	^18^F-FDG-Rhodamine	^18^F-FBnTP	^18^F-FTTP	^18^F-mFMBTP	^18^F-Flurpiridaz	^18^F-Fmpp2
Class	ammonium cation	phosphonium cation	phosphonium cation	phosphonium cation	MC-1 inhibitors	MC-1 inhibitors
Charge	cationic	cationic	cationic	cationic	neutral	neutral
Log P	−1.64 ± 0.03	--	1.78 ± 0.05	1.05 ± 0.01	--	1.73 ± 0.05
RCY (%)	97.0 ± 1.9 (based on ^18^F-FDG)	62 ± 1.4 (NDC)	10–15 (EOS)	50 (DC)	25 (DC)	58 ± 7.1 (DC)
Heart Uptake (%ID/g)	11.24 ± 1.97 (rat)	--	1.51 ± 0.04 (rat)	27.39 ± 1.46 (mice)	9.5 ± 0.5 (mice)	27.15 ± 3.58 (mice)
Heart/Liver Ratio	21.2 (rat)	1.2 (dog)	8 (rat)	4.84 (mice)	8.3 (mice)	3.96 (mice)
Heart/Blood Ratio	28.10 (rat)	16.6 (dog)	75.5 (rat)	23.82 (mice)	--	10.29 (mice)
Time point (min) *	--	60	30	30	60	30
Current status	rats	dogs	rabbits	dogs	Clinic trial (Phase III)	pigs
References	26	21, 32, 37	39	44	53	62

* Time point means the time point of heart uptake and heart/liver ratios in Table 1.

## Abbreviations

CAD	coronary artery disease
SPECT	single-photon emission computed tomography
MPI	myocardial perfusion imaging
PET	positron emission tomography
MC-1	mitochondrial complex-1
SUV	standardized uptake value
AcChE	acetylcholinesterase
p.i.	post injection
PEG	polyethylene glycol
NDC	non-decay-corrected
DC	decay-corrected
^18^F-FTMA	4-^18^F-fluorotri-*N*-methylanilinium
^18^F-FERhB	2-^18^F-fluoroethylrhodamine B
^18^F-FDG-rhodamine	^18^F-fluoro-2-deoxy-d-glucose-rhodamine
BODIPY	Boron-dipyrromethene
^18^F-2	10-(4-(trimethylammonio)phenyl)-5-fluoro-5-^18^F-fluoro-1,3,7,9-tetramethyl-5*H*-dipyrrolo-[1,2-c:2′,1′f][1,3,2] diazaborinin-4-ium-5-ide
^18^F-FBnTP	^18^F-fluorobenzyltriphenylphosphonium
^18^F-FTPP	(4-^18^F-fluorophenyl)triphenylphosphonium cation
^18^F-FPTP	(^18^F-fluoropentyl)triphenylphosphonium cation
^18^F-FHTP	(6-^18^F-Fluorohexyl) triphenylphosphonium cation
^18^F-FETMP	(2-(2-^18^F-fluoroethoxy)ethyl) tris(4-methoxyphenyl)phosphonium cation
^18^F-FMBTP	4-(^18^F-fluoromethyl)benzyltriphenylphosphonium
^18^F-mFMBTP	(3-(^18^F-fluoromethyl) benzyl)trisphenylphosphonium
^18^F-FDHR	^18^F-fluorodihydrorotenone
^18^F-flurpiridaz (BMS-747158-02)	2-*tert*-butyl-4-chloro-5-[4-(2-^18^F-fluoroethoxymethyl)benzyloxy]-2*H*-pyridazin-3-one
^18^F-FP1OP	2-*tert*-butyl-4-chloro-5-(4-(2-^18^F-fluoroethoxy))benzyloxy-2*H*-pyridazin-3-one
^18^F-FPTP2	2-*tert*-butyl-4-chloro-5-((6-((4-(2-^18^F-fluoroethyl)-1*H*-1,2,3-triazol-1-yl)methyl)-2-pyridinyl)methoxy)-3(2*H*)-pyridazinone
^18^F-Fmp2	4-chloro-2-*tert*-butyl-5-[2-[[1-[2-[^18^F]fluoroethyl]-1*H*-1,2,3-triazol-4-yl]methyl]phenyl-methoxy]-3(2*H*)-pyridazinone
[^18^F]Fmpp2	4-chloro-2-*tert*-butyl-5-[2-[[1-[2-[2-[^18^F]fluroethoxy]ethoxymethyl]-1*H*-1,2,3-triazol-4-yl]-methyl]phenylmethoxy]-3(2*H*)-pyridazinone
